# Use of Magnetic Resonance Imaging for Visualization of Oral Dosage Forms in the Human Stomach: A Scoping Review

**DOI:** 10.1021/acs.molpharmaceut.3c01123

**Published:** 2024-03-05

**Authors:** Tejal Akbar, Pavel Gershkovich, Konstantinos Stamatopoulos, Penny A. Gowland, Snow Stolnik, James Butler, Luca Marciani

**Affiliations:** † Nottingham Digestive Diseases Centre and National Institute for Health Research (NIHR) Nottingham Biomedical Research Centre, Nottingham University Hospitals NHS Trust and University of Nottingham, Nottingham NG7 2UH, U.K.; ‡ School of Pharmacy, University of Nottingham, Nottingham NG7 2RD, U.K.; § Drug Product Development, GSK R&D, Ware, Hertfordshire SG12 0GX, U.K.; ∥ Sir Peter Mansfield Imaging Centre, School of Physics and Astronomy, University of Nottingham, Nottingham NG7 2QX, U.K.

**Keywords:** in vivo, stomach, oral dosage forms, magnetic resonance imaging, MRI, oral drug delivery, dosage form disintegration, fasted stomach, fed stomach, MRI contrast markers, MRI contrast agents

## Abstract

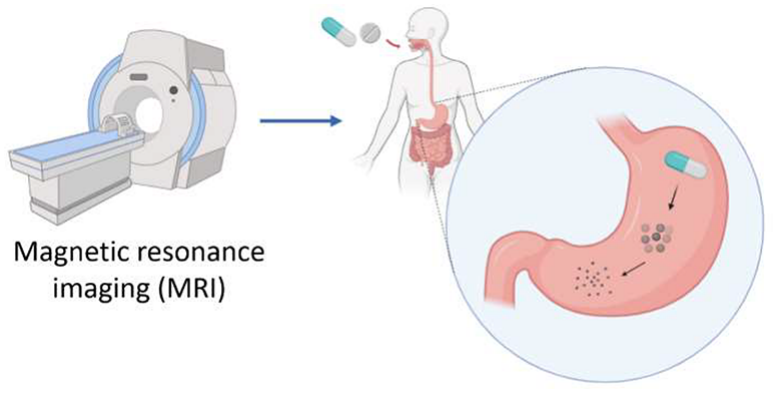

Oral dosage forms are the most widely and frequently used formulations to deliver active pharmaceutical ingredients (APIs), due to their ease of administration and noninvasiveness. Knowledge of intragastric release rates and gastric mixing is crucial for predicting the API release profile, especially for immediate release formulations. However, knowledge of the intragastric fate of oral dosage forms *in vivo* to date is limited, particularly for dosage forms administered when the stomach is in the fed state. An improved understanding of gastric food processing, dosage form location, disintegration times, and food effects is essential for greater understanding for effective API formulation design. *In vitro* standard and controlled modeling has played a significant role in predicting the behavior of dosage forms *in vivo*. However, discrepancies are reported between *in vitro* and *in vivo* disintegration times, with these discrepancies being greatest in the fed state. Studying the fate of a dosage form *in vivo* is a challenging process, usually requiring the use of invasive methods, such as intubation. Noninvasive, whole body imaging techniques can however provide unique insights into this process. A scoping review was performed systematically to identify and critically appraise published studies using MRI to visualize oral solid dosage forms *in vivo* in healthy human subjects. The review identifies that so far, an all-purpose robust contrast agent or dosage form type has not been established for dosage form visualization and disintegration studies in the gastrointestinal system. Opportunities have been identified for future studies, with particular focus on characterizing dosage form disintegration for development after the consumption food, as exemplified by the standard Food and Drug Administration (FDA) high fat meal.

## Introduction

Oral administration is a preferred and well-established route for active pharmaceutical ingredient (API) delivery, with capsules and tablets being popular choices due to the ease of self-administration, their noninvasiveness, and relatively high patient compliance.^1,2^ In 2023, the Food and Drug Administration’s (FDA) Centre for Drug Evaluation and Research (CDER) approved 55 new molecular entities and new therapeutic biological products.^3^ A significant proportion, 44%, were designed to be orally administered. Delivery of an API to the systemic circulation via oral administration is through API release within the gastrointestinal tract (GI), often through processes of disintegration and deaggregation. Disintegration is the mechanical break up of a dosage form into smaller pieces, resulting in API release. For a hard shelled or soft gel capsule, this may appear as a change in the capsule shape or rupture of the shell itself, resulting in a release of the capsule contents. The disintegration of tablets manufactured by direct compaction is a complex process, in part dependent on the physical changes that occur during the compression process. These include elastic deformation, plastic deformation, fragmentation of particles, as well as the formation of interparticle bonds. For disintegration to occur, a force greater than the interparticle forces and bonds must be applied. The disintegration process of a compacted tablet is often initiated by liquid penetration, known as wicking. This is often a prerequisite to initiate other mechanisms of disintegration. The liquid penetrates the tablet through the pores in the microstructure. The most common disintegration mechanism is the omnidirectional enlargement of particles, commonly identified as swelling.^4^ Strain recovery, commonly known as shape recovery, is where the particles enlarge unidirectionally. Disintegration can additionally take place, whereby the excipients dissolve from the pore walls, known as the dissolution mechanism of disintegration. These mechanisms cause interruption of particle bonds, which results in the disintegration or break up of the compacted tablet. As the tablet breaks into smaller pieces through disintegration, the surface area available for dissolution increases, resulting in faster API release.^5^ Subsequently, dissolution of the API in gastrointestinal fluid permits delivery to the mucosa within the small intestine where it can be absorbed into the systemic circulation. Knowledge and understanding of key aspects of gastrointestinal fate of an oral dosage form include intragastric release rates, gastric mixing, food effects, and gastric transit times.^6^ These are often crucial for predicting the API release profile, especially for immediate release formulations.^7^ These factors are modified when the stomach is in a fasted or fed state.

*In vitro* models such as the TNO gastrointestinal model (TIM, TNO Quality of Life, Zeist, The Netherlands),^8^ GastroDuo,^6^ and standard pharmacopeia USP disintegration and dissolution tests (British Pharmacopoeia 2024 Appendix XII A. Disintegration)^9^ play an important role in formulation design and development. However, discrepancies have been observed between standard pharmacopeia *in vitro* and *in vivo* disintegration times,^10^ often made greater by the postprandial state.^11^ Abrahamsson et al. have shown that there is a food induced delay in disintegration and dissolution times of immediate release tablets *in vitro* and in the stomach of dogs.^12^ This was also demonstrated by the use of capsule endoscopy in beagle dogs, whereby it was shown that the *in vivo* tablet disintegration time was longer compared to the tablet disintegration *in vitro*.^13^ The discrepancy between *in vitro* and *in vivo* disintegration times shows that models have been unable to simulate the continuously evolving composition and mechanical processes of the postprandial stomach. Studying the fate of a dosage form *in vivo* is a challenging process, usually requiring the use of invasive methods such as intubation. Noninvasive, whole body imaging techniques can however provide unique insights into this process.

To date, a variety of whole-body imaging techniques such as gamma scintigraphy, magnetic marker monitoring (MMM), and magnetic resonance imaging (MRI) have been used to visualize and examine the fate of oral dosage forms in the stomach in the fasted and postprandial state.^14^ Senekowitsch et al.^15^ recently described the importance of *in vivo* techniques to support drug development in both academic and industrial settings.

Gamma scintigraphy has been an established and widely used method to visualize dosage forms *in vivo*.^16^ The dosage forms are labeled with γ radiation emitting radiopharmaceuticals before administration. The labeling of the dosage forms with the radioisotope can be performed in liquid, solid, and gaseous states. This is advantageous as the state of the dosage form can remain unchanged and poses no restrictions on the types of dosage forms which can be monitored.^14^ A comprehensive understanding of the fate and behavior of drug delivery systems in the gastrointestinal tract was introduced in 1976 by Casey^17^ and in 1981 by Hardy and Wilson.^18^ Knowledge gained from gamma scintigraphy studies has assisted with dosage form optimization, and quantification of the effects of formulation variables such as density, viscosity and coating on the transit of oral drug formulations.^19^ Studies using gamma scintigraphy have furthermore investigated the complex relationship between dosage form and food.^16^ It has been used to demonstrate that the gastric emptying and motility patterns differ depending on whether the stomach is in a fasted or fed state. The type and size of the dosage form has been identified to influence these processes.^20−22^ Gamma scintigraphy has additional benefits. For instance, it can facilitate gastric emptying studies in a seated or semireclined position. This provides the opportunity to obtain data in a more physiologically seated position. Nevertheless, gamma scintigraphy has some inherent limitations. It exposes the participants to ionizing radiation^23^ which can pose limitations when repeating studies for research purposes. Gamma scintigraphy does not have good temporal resolution and does not provide images of the anatomy, only a signal from the radiolabel, which limits in turn dosage form localization within the gastrointestinal organ itself and within the intraluminal food and drink matrix. Discrimination between the solid dosage form and liquid meal can be performed using two independent labels. Examples include technetium for dosage form location and indium for fluid labeling.

Magnetic marker monitoring (MMM) is an alternative noninvasive imaging technique. A solid dosage form is magnetically labeled with the addition of small amounts of ferromagnetic materials such as black iron oxide. After ingestion, its magnetic dipole field is recorded using a superconducting quantum interference device (SQUID) measurement device.^24^ The magnetic dipole is generated by aligning the magnetic orientations of the individual magnetic particles by magnetization prior to administration. The location and magnetic moment are estimated from the recorded data by fitting techniques. MMM enables a temporal resolution of a few milliseconds and a three-dimensional spatial resolution in the range of a few millimeters.^25^ It has been used to determine the gastrointestinal transit characteristics of both disintegrating and non-disintegrating solid oral dosage forms. In contrast to gamma scintigraphy, MMM is radiation free. However, it does not allow anatomic referencing and requires costly specialized equipment which is not generally available. In some cases, magnetically shielded rooms may be required, and it is only able to detect one single dipole.^15^

By contrast, magnetic resonance imaging (MRI) has become an increasingly popular and powerful imaging tool used for visualization and study of pharmaceutical processes within the gastrointestinal tract.^26^ MRI is able to acquire cross-sectional images of the body with excellent soft tissue contrast, in real time and with a large field of view.^27^ MRI is highly suited to imaging of the GI tract, as it can differentiate between solid and liquid components of ingested meals and intragastric gas. It has supported testing of new technologies for tailoring drug release^28−30^ and allowed detailed information on drug and food interactions to be collected.^8^ Drawbacks of MRI include high instrumentation costs and unsuitability for specific patient groups, such as those with metal implants. Patients living with obesity may not fit comfortably in smaller bore conventional MRI scanners, and those with claustrophobia may find MRI challenging. Conventional MRI images are taken using a horizontal bore scanner and in a lying down position. This may not represent entirely the physiological fate of an oral dosage form taken while maintaining an upright position. The size, shape, and position of the stomach is known to vary depending on the posture of the individual and on the volume of stomach content.^31^ The advent of open configuration magnets^32^ facilitates imaging in a sitting position and could overcome this limitation. Yet, the open configuration results in a less homogeneous magnetic field. The open design magnets operate also at a lower field strength and with different magnetic field gradient designs compared with conventional MRI scanners, resulting in an overall decreased image quality. In cases where the detection of smaller objects or small signal changes is required, an open confirmation may not be appropriate, even though the configuration may allow physiological conditions to be more closely represented.

Based on the considerations above, this scoping review aimed to identify and synthesize available literature on MRI studies that have visualized gastric transit of all oral dosage forms *in vivo* for healthy human intragastric processes. The scoping review aimed to identify gaps for further research in this field, with the intention of identifying knowledge that may help close the difference between *in vitro* models and *in vivo* dosage form behaviors.

## Materials and Methods

### Search Strategy and Protocol

A scoping review was performed in accordance with the Preferred Reporting Items for Systematic Reviews and Meta-Analyses (PRISMA) extension for scoping reviews (PRISMA-ScR): checklist and explanation.^33^

The following question was proposed: “How has MRI been used to visualize solid oral dosage forms in the fasted and fed healthy human stomach?”. The protocol for the conduct of the scoping review was published on the Open Science Framework (OSF) registry on the 8th of March 2023 (https://doi.org/10.17605/OSF.IO/X2EDP.) prior to commencing the review. Once the review began, the search criteria was extended to include not only the stomach but also the gastrointestinal environment. Many of the identified studies included imaging in both the stomach and other areas of the gastrointestinal system. Inclusion of these papers revealed additional valuable data.

The search criteria were formulated based on the Population, Intervention, Comparator, Outcome (PICO) framework.^33^ A comparator was not considered and was omitted from the search criteria. Only population, invention, and outcome (PIO) data were considered. Healthy human subjects were identified as the population of interest. The intervention considered was MRI, and the outcome measured was the visualization of solid dosage forms within the stomach, in both fasted and fed states.^34^

### Database Search and Inclusion/Exclusion Criteria

The key objective of the search was to locate published peer reviewed literature. An initial search of Google Scholar was undertaken to identify articles and keywords in the research area. Keywords and phrases contained in the titles and abstracts of relevant articles and the indexed medical subject headings (mesh) terms used to describe the articles were used to develop a full search strategy. The search strategy and all identified keywords and index terms were adapted for each included database. The Medline (Ovid), Embase (Ovid), and Web of Science databases were searched. A supplementary search strategy was created for Google Scholar, whereby the first 500 articles in the results were considered. Sources of unpublished studies and gray literature were additionally searched using Google Scholar. Only studies published in English were included with no limitation placed on the study date. Reviews or systematic literature reviews and abstracts were not considered.

No restrictions were placed on population demographics, for example, age or sex. Only healthy human studies were considered, and studies that mentioned disease were excluded. Animal studies were additionally excluded from the search because animal research studies, particularly those relating to pharmaceuticals, have shown little correlation with human experience.^30^ Studies using MRI only were included. Studies performed with the use of magnets or associated techniques such as MMM, magnetic biosusceptometry, and magnetic spectroscopy were excluded from the search. Orally administered solid dosage forms were considered; both immediate release and modified time release were included. The inclusion and exclusion criteria are summarized in Table 1. The keyword search was extended beyond the stomach to include the gastrointestinal system to capture a full representation of the subject area, with an emphasis on the fasted and fed state stomach.

**Table 1:** Summary of the Inclusion and Exclusion Criteria Used for Database Searches

inclusion criteria	exclusion criteria
all ages	animal studies
all sexes	reviews or systematic literature reviews
healthy human studies	abstracts
MRI studies	studies performed with use of magnets such as MMM, magnetic biosusceptometry, and magnetic spectroscopy
orally administer dosage forms	studies published in a language other than English
studies investigating the gastrointestinal system	

### Study/Evidence Selection Process

**Figure 1:**
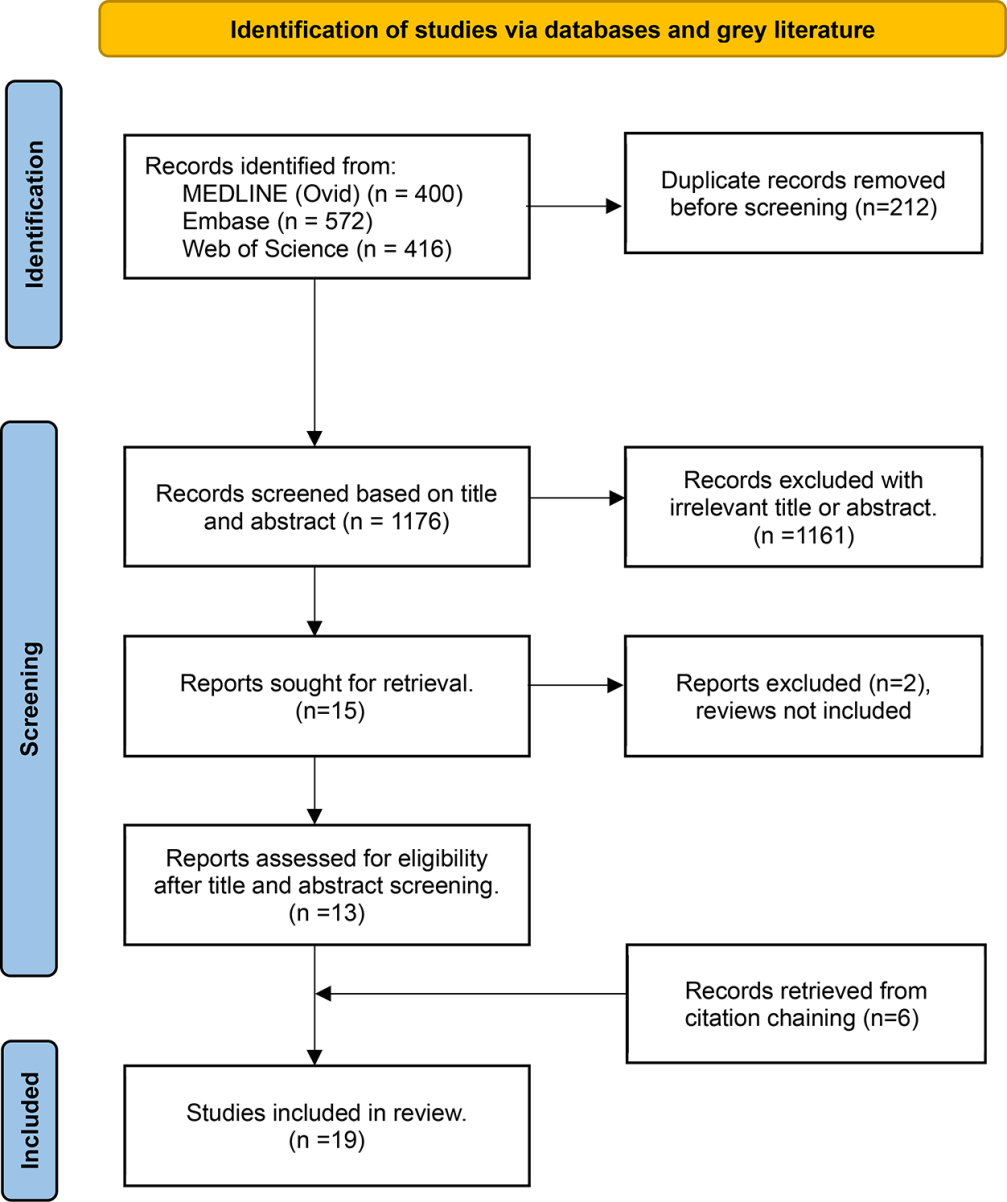
PRISMA 2020 flow diagram for scoping reviews showing the process used to identify the published literature included in the scoping review.^35^

All identified citations from all databases were collated into the software management tool Endnote 20 (Clarivate Analytics, Philadelphia, PA, USA), where duplicates were removed (Figure 1). Titles and abstracts were screened in full by another independent reviewer for assessment against the inclusion criteria for the review. The full text of selected citations was assessed in detail against the inclusion criteria. Disagreements that arose between the reviewers at each stage of the selection process were resolved through discussions. Further to the papers, cross citations, author searches, and where the paper was referenced were searched (Figure 1).

### Data Extraction

Data was extracted in a tabular form using Microsoft Excel (Microsoft Corporation, 2018). Research aims, practicalities, and study parameters were extracted such as inclusion/exclusion criteria of the participants, study restrictions including fasting protocols, and how and when the dosage form was administered. Specific information regarding dosage forms was extracted, such as the dosage form size and composition and the contrast agent used in the dosage form. Importantly, any definitions the researchers used to identify and quantify disintegration or dissolution in the images were extracted. The MRI imaging parameters were extracted such as imaging times, type of sequence employed, T1 or T2 weighting, and echo and repetition times. The conclusions of each study were noted. The scoping review follows a narrative approach. Results are synthesized into a narrative summary.

## Results

### Data Synthesis and Discussion

In total, 19 published papers were included in the review. All were published between 2001 and 2024 (Table 2).

**Table 2:** Summary of Data Extracted from 19 Published Papers Identified in the Review

author, year	participant imaging position	fasted or fed stomach condition	dosage form type	contrast agent
H. Faas (2001)^36^	lying with the left side elevated at an angle of 30° to the horizontal	fed, liquid meal (400 kcal); solid meal (795 kcal)	capsule	Gd-DOTA^a^
H. Faas (2002)^37^	lying with the right side elevated at an angle of 30° to the horizontal; supine	fed, homogeneous meal (636 kcal); particulate meal, (657 kcal); hamburger meal (650 kcal)	capsule	Gd-DOTA
A. Steingoetter (2003)^38^	sitting (upright)	fed, semisolid test meal (922 kcal)	tablet	Fe_3_O_4_ and Gd-DOTA
A. Steingoetter (2003)^27^	sitting (upright)	fed, hamburger meal (921.9 kcal); cheese meal (734.6 kcal); pasta meal (934.9 kcal)	tablet	Fe_3_O_4_
C. Schiller (2005)^39^	supine	fasted and fed state: meal of noodle soup, chicken with rice, mixed vegetables, fruit yoghurt (803 kcal)	capsule	gel based on glycerol, gelatin, and water, solid triglyceride
L. Kagan (2006)^30^	supine	fed, 280 kcal meal	capsule	Fe_3_O_4_
M. Knörgen (2010)^40^	supine	fed, semisolid meals	pellets	Fe_3_O_4_
L. Curley (2017)^41^	supine	fasted state dosed with 2 × 250 mL of water	caplets and tablets	none
M. Sager (2018)^42^	supine	fasted and fed state: breakfast (964 kcal)	ice capsule	none
M. Grimm (2019)^43^	supine	fasted, dosed with 240 mL of water	capsule	dried and sugared pineapple pieces
M. Sager (2019)^44^	supine	fasted, dosed with 240 mL of water	capsule	Fe_3_O_4_
M. Sager (2019)^45^	supine	fed, light breakfast (500 kcal)	capsule	Fe_3_O_4_
M. A. El-Aziz (2021)^29^	supine	fed, low-calorie meal	tablet	Fe_3_O_4_
A. Rump (2021)^46^	supine	fasted, dosed with 240 mL of water	capsule	Fe_3_O_4_ and hibiscus tea powder
A. Rump (2022)^47^	supine	fasted, dosed with 240 mL of water	capsule	Fe_3_O_4_
S. Sulaiman (2022)^48^	supine	fasted, dosed with 240 mL of water	capsule	olive oil
A. Rump (2023)^28^	supine	fasted, dosed with 240 mL of water	minitablets encapsuled in capsules	Fe_3_O_4_
M. Grimm (2023)^49^	supine	fed, light meal (500 kcal)	capsule	Fe_3_O_4_
I. Seoane-Viaño (2024)^10^	supine	fasted, dosed with 250 mL water	torus shaped tablets (printlets)	MnCl_2_

a Gadolinium tetraazacyclododecane tetraacetic acid.

The following discussion provides a critical evaluation of the benefits, capabilities, and limitations of using MRI for dosage form visualization in the gastrointestinal tract. The capabilities of using MRI and how it has been used to quantify and assess the gastric environment in the fasted and fed stomach are discussed. The roles of dosage forms in achieving these results are explored. The development of contrast agents for dosage for visualization is explored. Suggestions are provided for future studies, and knowledge gaps have been identified for development and advancement in the field. It is also worth noting that the majority of the papers reviewed originated from just two research groups in Germany (47% of the total) and Switzerland (26% of the total), so this may bring some “group bias” to the data extracted.

### MRI Contrast Agent Development

The most common dosage forms comprise dry material, which by itself does not provide MRI signal. Imaging small, “dark” objects in the body is particularly difficult. Curley’s^41^ feasibility study, performed with an unmodified paracetamol formulation, concluded that without the addition of a contrast agent, a dosage form may become undetectable once it has passed from the stomach to the duodenum and may not even be detectable in the stomach after meal intake.

A common trick exploited in the papers reviewed to overcome this problem was to modify the dosage form to make it more MRI visible, thereby helping detection. This was done by adding either paramagnetic/ferromagnetic materials that can cause an image artifact bigger than the dosage form or a material with a high fat content to help differentiate it from the surroundings such as water, food materials in the gut lumen, or tissue such as gut walls. MRI contrast agents are materials that can modify or distort the properties of the magnetic field around the dosage form and of the surrounding environment in which they are placed, causing signal changes that can be more easily detected in the images. When a contrast agent is dissolved in water, it modifies the relaxation times of the hydrogen protons of the water molecules. T1 (spin–lattice relaxation) and T2 (spin–spin relaxation) are two key time constants of the imaging process, and these two parameters reflect how quickly the water protons return to their equilibrium state after a radiofrequency excitation during the MRI imaging process. Contrast agents that primarily shorten the T1 relaxation time when dissolved in water are commonly referred to as “positive” contrast agents as they cause T1 signal enhancement or a “bright” appearance on a T1 weighted MRI image. Examples include manganese, copper, and gadolinium. Negative contrast agents, however, shorten primarily the T2 relaxation time when they are dissolved in water. They cause a darkening in a T2 or T2* weighted image. The extent and influence of contrast agent depends on the material’s magnetic susceptibility. This is a measure of the extent to which it becomes magnetized when placed in an external magnetic field, i.e., the field in an MRI scanner. For example, gadolinium, copper, and manganese are paramagnetic materials and have some effect, whereas iron is ferromagnetic, causing a large effect on the magnetic field in which its placed.

A wide range of different materials have been tested in these review studies, including gels, magnetite, dried and sugared pineapple, manganese chloride tetrahydrate (MnCl_2_), Gd-DOTA, oil, and hibiscus tea (Table 2).

Schiller^39^ developed non-disintegrating capsules constructed from pellets surrounded by a solid triglyceride material. The pellets were constructed from a gel based on glycerol, gelatin, and water. The watery gel pellets appeared white in the T2 weighted MRI images, in contrast to the surrounding solid triglyceride, which appeared dark. This dosage form was created to exploit the differences between fat and water for it to be visible in an MRI image. This novel method used the addition of one, two, or three pellets to identify time of capsule administration. The study demonstrated that the fluid available to dosage forms varies along the intestine and in the fasted and fed state. This inhomogeneous distribution was said to likely contribute to the individual variability of drug release and absorption in modified release dosage forms.

Magnetite, Fe_3_O_4_, was used as a contrast agent in 10 of the 19 papers reviewed. It is a ferromagnetic material whose primary component is iron oxide, containing equal amounts of iron(II) and iron(III). Magnetite, in addition to being superparamagnetic also acts as a negative contrast agent. It shortens T2 and T2* relaxation times, producing a darkening in a T2 weighted MRI image. Magnetite susceptibility features are strongly visible in T2 weighted images rather than in T1 weighted images. This is an important methodological consideration for future studies using MRI and selection of MRI suitable sequences. Development of MRI protocols and imaging sequences reflect the choice of contrast agent and study purpose to enable adequate dosage form visualization. T2 weighted imaging sequences such as a HASTE (Half Fourier Single-shot Turbo spin–Echo) or TRUFI (True Fast Imaging with Steady-State Free Precession) sequences have been identified as popular choices in this review where anatomical information is required, and magnetite was chosen as the contrast agent. The development of new study protocols for further studies would require consideration of what information is required from the study to determine the appropriate imaging sequences and parameters for use, i.e. higher resolution for smaller dosage forms.

**Figure 2:**
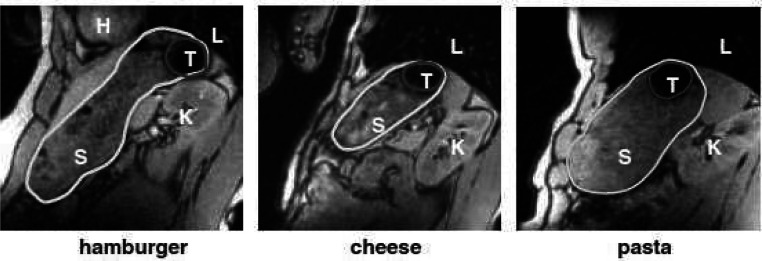
Sagittal MRI images of the stomach taken in an upright seated position. Images show a feature caused by a magnetite tablet present in three separate meals: hamburger, cheese, and pasta meals. Anatomical landmarks are indicated by capital letters: H, heart; K, kidney; L, lung, S, stomach; and T, tablet. Image reproduced with permission from ref (38). Copyright 2003 John Wiley & Sons Ltd.

Steingoetter^38^ was the first to evaluate the use of magnetite as a contrast agent in powder form and produced a tablet through direct compression. A magnetite concentration of 1% of the tablet volume provided a sufficiently large stable signal drop out feature for use in an *in vivo* study. In a study conducted in an open configuration MRI scanner, susceptibility features were observed within either a hamburger, cheese, or pasta meal and could be distinguished from the intragastric air. The artifacts caused by magnetite were distinguishable from intragastric signal voids, such as air or solid pieces of meal. The open experimental set up allowed volunteers to freely move between measurements, ideal for long study periods. Figure 2 shows a sagittal MRI image of the stomach showing a clearly identifiable susceptibility feature from magnetite within various meals.^38^

Magnetite was popular for several applications for use as an MRI contrast agent due to its low toxicity and availability in powder form. The superparamagnetic properties of magnetite result in large susceptibility features without the requirement for interaction or dissolution in water. This in combination with its robust nature made it straightforward to incorporate into both capsule shells and tablet dosage form types. This powerful feature was ideal for testing and developing gastroresistant capsules,^30,45,47^ where disintegration of the capsule shell was delayed until it reached the intestine.

Knörgen^40^ in 2010 recognized a requirement to image alternative types of small dosage forms such as pellets and used magnetite as a contrast agent. They considered the choice of MRI imaging sequence to be important for the susceptibility artifacts. They developed a novel post-processing method to create susceptibility maps, thus enabling detection of magnetite pellets within a meal. The method was able to detect pellets both *in vitro* and *in vivo* within a variety of heterogeneous foods. However, a standard model “fit” could not be developed as a large intersubject variability was reported. It was noted that the main drawback of MRI for this purpose was from physiological boundaries such as stomach contractions and cycle and peristaltic waves leading to images having to be acquired within a breath hold.

The use of magnetite was explored for the determination of the disintegration site and time for dosage forms, predominantly capsules. However, in several studies,^28,43,45,47^ problems determining exact disintegration site and time were reported due to the large size of the susceptibility feature of magnetite. This limitation was addressed by Grimm^49^ in a study where they made a clear distinction not to define a capsule rupture, but a process of “dispersion”, defined as the appearance of several small artifacts or a change in the size or the geometry of the artifact. They observed that small leakages in capsules may not necessarily lead to the complete dispersion of the capsule filling. The process of dispersion was highlighted as a step of disintegration but not disintegration itself. In the study of Lonza Capsugel Next Generation Enteric Capsules,^47^ this was also echoed. It was noted that a change in spatial distribution of the magnetite artifact suggested a disruption of the capsule integrity, and therefore the start of the disintegration time may be overestimated. The use of a pharmacokinetic marker such as caffeine in saliva,^44^ was employed in several studies and it provided an additional method to improve the accuracy of establishing disintegration time of the capsule shell.

Rump^47^ highlighted that the viscosity of the surrounding media must be low enough for the magnetite to distribute once released from the capsule to be observed by MRI. This needs to be kept in consideration when evaluating if magnetite would be appropriate for use in studies used to determine dosage form disintegration. The surrounding environment in which the dosage form is located could be a limiting factor to allow sufficient distribution of magnetite, for example, a postprandial stomach containing a highly viscous meal or fatty meal. Another important consideration is that after the release from a dosage form, magnetite is rather insoluble in the gastric environment. Therefore, the susceptibility feature produced by magnetite could persist regardless of API and excipient dissolution. This, in turn, could decouple image features from true dissolution of the API. This insolubility of iron was noted when designing 3D laser printed tablets.^10^ It was considered a limiting factor for the determination of dosage form disintegration and, therefore, not used in the *in vivo* study for those purposes.

As a result of this review, it has been identified that MRI has been used to validate disintegration time and location; it has however not been used to directly visualize the disintegration process of a dosage form *in vivo*, in capsule or tablet form. This further work may help to identify the limitations and capabilities of MRI imaging. Tablets may be appropriate to show hydration, ideal for MRI which images water. The characterization of the processes of disintegration may help to bridge the gap between *in vitro* and i*n vivo* measurements. Development and identification of a suitable contrast agent and imaging sequence will be key for this.

Alternative contrast agents used in the papers reviewed included paramagnetic gadolinium compounds, manganese chloride tetrahydrate, dried and sugared pineapple, and hibiscus tea. Safety concerns regarding the use of gadolinium as a contrast agent,^34,48^ when not needed for scans prescribed for a medical reason, now discourage the use of this agent for research studies. The latter two contrast agents listed exhibit elevated quantities of manganese ions, leading to a signal increase in a T1 weighted MRI imaging sequence and a decrease in a T2 weighed MRI image. These positive contrast agents need to interact or dissolve with water for an effect on the relaxation times to occur. When they are in dry form, they are still difficult to detect in an MRI image and, therefore, may fall short as a suitable contrast agent in comparison to magnetite. This is reflected in the limited use of these materials for evaluation of gastroresistant capsules where no water interaction in the upper GI tract is expected. However, to help characterize the disintegration behavior of capsules, dried and sugared pineapple was evaluated by Grimm.^41^

**Figure 3:**
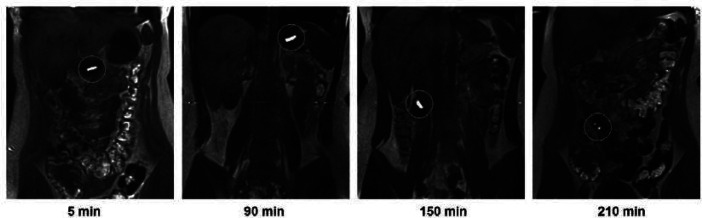
T1 weighted MR images taken in a supine position of a pineapple filled capsule at 5 min (stomach), 90 min (stomach), 150 min (ileum), and 210 min (disintegrated in ileum) after administration in a fasted state. Image reproduced with permission from ref (43). Copyright 2019 Elsevier.

**Figure 4:**
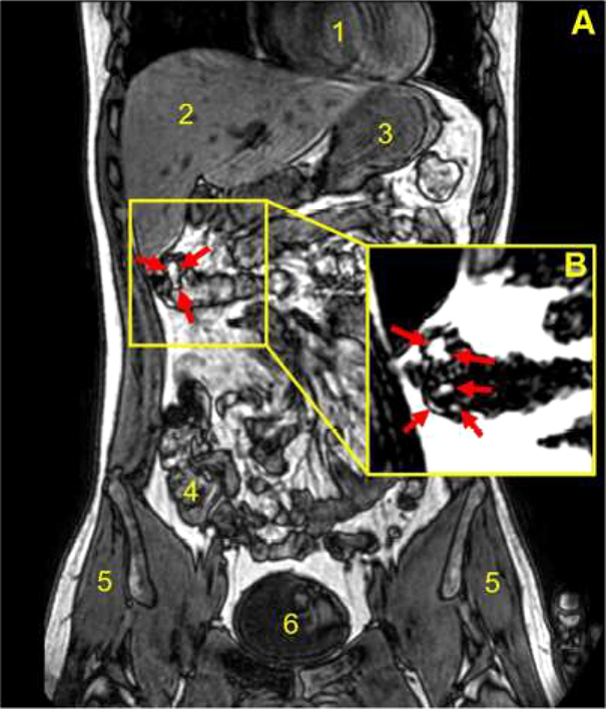
Coronal, fat, and water out of phase MRI image taken in a supine position. The deformation and uneven filling of an oil filled coated capsule is shown. The inset (B) is from the corresponding fat only MRI image, capturing oil leaking out of the capsule into the colon, indicated by the red arrows. Anatomical landmarks are given for heart (1), liver (2), stomach fundus (3), transverse colon (4), gluteus medius muscle (5), and bladder (6). Image adapted with permission under a Creative Commons CC BY license from ref (48). Copyright 2022 MDPI.

The positive contrast from the pineapple was clearly visible in the T1 weighted images (Figure 3) and T2 weighted images that were taken for anatomical imaging. The varying moisture content of the pineapple proved problematic for sample continuity. If pineapple was to be integrated as a contrast agent into alternative dosage forms such as compressed tablets for future studies, this would require further consideration. In the same manner as magnetite, in this study, the first point of disintegration of the capsule shell splitting was difficult to observe. A study by Sulaiman^48^ was the only study identified in this review that imaged directly the loss of integrity of a capsule shell filled with olive oil as a MRI fat-visible marker (Figure 4).

Oil as a contrast agent, however, would pose several limitations, as it may alter the release of the capsule, making it unviable to use in capsules designed for powder or dry contents only. Additionally, oil would not be a viable option for use in compressed tablets due to its liquid nature.

**Figure 5:**
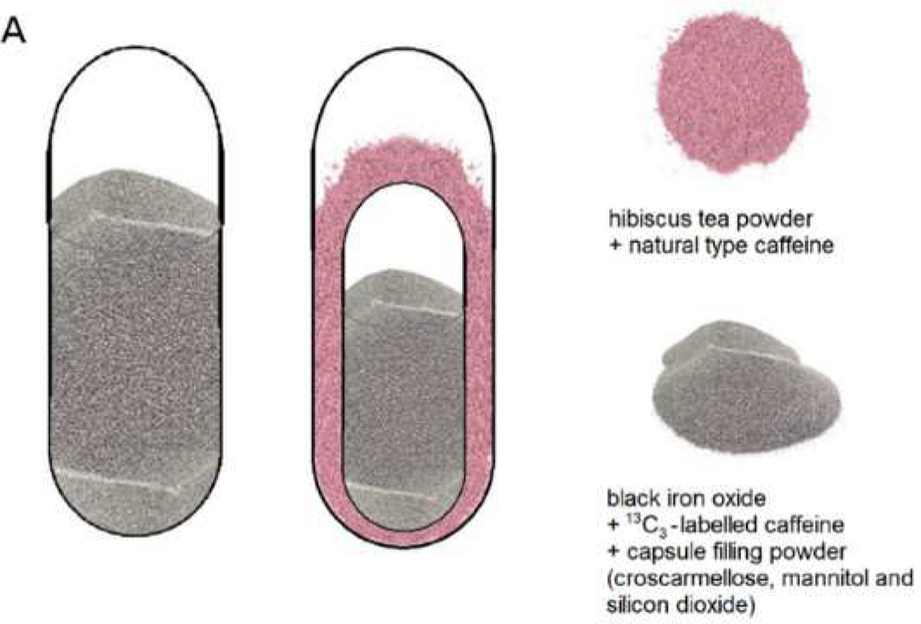
Composition of the single capsules (left) filled with black iron oxide and of the size 3 capsule in size 00 capsule formulations (right) filled with a combination of hibiscus tea and black iron oxide. Image adapted with permission under a Creative Commons CC BY license from ref (46). Copyright 2021 MDPI.

Evaluation of the contrast agents in this review showed that no material was used successfully in both a capsule and tablet form for imaging disintegration, in either a fasted or fed stomach, using MRI. Finding a more widely applicable contrast agent is challenging, as an ideal agent must not change the physical properties of the dosage form and must be available in several forms such as powder and liquid. Visualization of the first point of disintegration is of importance to assess the bioavailability of the dosage form to the systemic circulation and therefore, in many cases, its success. When imaging using MRI, several factors must be considered in choosing the appropriate contrast agent; it must be visible in the image and differentiate itself from the environment. An appropriate imaging sequence must be chosen, and it must interact in the appropriate way to be visible. Several attempts have been made to identify an “ideal” contrast agent, but such a contrast agent is not yet available, and different materials may be required for different purposes, maybe even combined. For example, Rump^46^ used a combination of hibiscus tea and magnetite (Figure 5). In this study, *in vitro* measurements did not correlate with *in vivo* observations, and further development was required.

The addition of pharmacokinetic markers such as caffeine may also be attractive in instances where MRI might not provide the sensitivity required. A key next step for further enhancing the application of MRI studies would be to design a contrast agent that not only is able to quantify disintegration processes of the dosage form but also is able to provide information on what happens after disintegration: dissolution. No studies in this review have used dosage forms for imaging dissolution. The advent of 3D laser printing of dosage forms and the ability to vary porosity and therefore disintegration times through modification of laser scanning speed may be an alternative opportunity to explore. Further data will help to close the gap between *in vitro* models and *in vivo* processes.

As mentioned previously, not only will the contrast agent selection be an important factor, but also the type of dosage form itself. In this review, references to tablets, caplets, pellets, and laser printed torus shaped printlets were identified. In 12 out of the 19 papers reviewed, capsules or combinations of capsules, i.e., capsules in capsules (Lonza Capsules & Health Ingredients, Greenwood, SC)^46^ were utilized. Tablets have not been used as frequently although gastroretentive tablets have been successfully used to study their disintegration and distribution in the presence of food.^27,38^ This indicates that tablet behavior is a key area where there has been less research, and where there are opportunities for further research and development.

### Visualization of Dosage Forms Using MRI

Little variation in the type or field strength of MRI scanners used to study the dosage forms in the gastrointestinal system has been observed throughout the study period of 2001 to 2024. In many studies, either a 1.5T or 3T conventional horizontal bore scanner was used, in which a supine position is adopted by participants. Two studies^27,38^ in the review use an open configuration scanner as opposed to a conventional horizontal bore scanner. Steingoetter^27^ took advantage of the upright position to investigate gastroretentive floating tablets taken with a semisolid meals. The study was able to visualize the intragastric position of the tablet relative to the gastric contents which proved helpful for the understanding of the tablets’ intragastric pathway. If this investigation was performed in a supine position, the information may have differed due to the posture variation. The susceptibility artifact of magnetite present in the tablet was distinguishable from other intragastric signal voids such as air or solid pieces of meal.^27^ A recent paper by Seoane-Viaño,^10^ highlighted that a dosage form may be exposed to different gastric fluids and differing mechanical forces when adopting a supine imaging position compared to a sitting position. These factors should be considered when assessing the dosage form performance. The outcomes of the review show that there is a possibility for further investigation using open configuration scanners and imaging in an upright seated position, providing a physiologically representative capture of the processes inside the gastrointestinal system. The open configuration would enable participants to have the option of freely moving between measurements, providing opportunities for prolonged imaging studies. The open configuration allows participants to eat or drink within the scanner, allowing for these processes to be imaged in real time and for the effects of posture and gravity to be eliminated when moving from administration to the supine imaging position. Further investigations would benefit from comparing and contrasting images obtained in both upright and supine positions to determine how and if the data and imaging protocols differ in this alternate imaging position.

The unique ability of MRI to distinguish between fat and water has allowed the progression of imaging of dosage forms and food interactions. It is recognized that MRI is not solely limited to imaging of protons (^1^H), but it can image also other MRI active nuclei such as sodium (^23^Na) and fluorine (^19^F). For instance, Hanh^50^ performed successful real time intestinal tracking of ^19^F labeled capsules. However, this review focuses on MRI performed with protons.

The following section provides an outline of how MRI has been used to progress this field. The capabilities of anatomical dosage form location and gastric environment qualification using MRI were exploited by Faas^37^ in a study, where it was observed that a meal and contrast agent were mixed more homogeneously in the antrum. They showed that a large part of the meal in the fundus was not accessible to the contrast agent. This work was further validated by Steingoetter,^27^ who showed that Gd-DOTA used as a contrast agent dispersed along the posterior gastric wall into the distal stomach bypassing most of the meal in the fundus. They observed that in certain circumstances, the order in which a dosage form and meal were ingested did not affect the order in which they were emptied from the stomach. They also observed that a dosage form may be emptied from the stomach before the meal, even when it is ingested after the food. The paper noted the contrast agent showed a preferential distribution from the fundus along the inner curvature of the stomach wall into the antrum, and there was no dependence on the liquid content of the meal. These studies showed the importance of characterizing the intragastric environment, something that has been possible since the advent of MRI. MRI has shown that the availability of water to the dosage form in the intestine is not uniform but is available in small pockets.^39^ It has been able to quantify that the type and composition of a meal play important roles in the disintegration and dissolution processes of a dosage form. These findings may help in adapting the *in vitro* standardized disintegration and dissolution tests to replicate the inhomogeneous conditions truly experienced by a dosage form, and thereby bridging the gap of predicting *in vivo* performance with *in vitro* testing in the presence of food. This review has highlighted that although interactions between test meals and dosage forms have been characterized, no studies with standard dosing tests to date have been performed with meals that are high in fat content, i.e., greater than 50% fat, containing 800–1000 kcal as outlined by the FDA.^51^ Further research in the fed state would enable a better understanding of food interaction^12,10^ and improved tailoring of gastric dosage form disintegration and drug release for immediate release formulations, especially as the importance of liquid availability was demonstrated using immediate release formulations of aspirin^4^ and fosamprenavir.^8^

## Conclusion

While previous reviews highlighted the role and importance that MRI plays in the pharmaceutical industry,^14,15^ we performed an in-depth critical review in the specific area of oral dosage form visualization *in vivo* using MRI. This scoping review has summarized current best practice and has identified possible contrast agents that may be used for dosage form visualization in MRI and outlined considerations and knowledge gaps for future studies. It is clear that as yet, an all-purpose robust contrast agent has not been established. However, opportunities have been identified for future studies, with particular importance on characterizing dosage form disintegration and studies characterizing dissolution.

We have highlighted the important role that MRI imaging can play in characterizing the dosage form behavior and the intragastric environment in which the dosage form is located. Knowledge gaps have been highlighted, for instance, in characterizing meals high in fat content. MRI imaging of dosage forms *in vivo* has the potential to lead to better *in vitro* and *in vivo* agreement for improved oral dosage drug development and ultimately may be used to enhance the ability to tailor drug release of oral dosage forms.

## Abbreviations

API: active pharmaceutical ingredient
MRI: magnetic resonance imaging
FDA: Food and Drug Administration
CDER: Centre for Drug Evaluation and Research
GI: gastrointestinal tract
MMM: magnetic marker monitoring
SQUID: superconducting interference device
PRISMA: Preferred Reporting Items for Systematic Reviews and Meta-Analyses
OSF: Open Science Framework
PICO: Population, Intervention, Comparator, Outcome
mesh: medical subject headings
Gd-DOTA: gadolinium tetraazacyclododecane tetraacetic acid
PK: pharmacokinetic
HASTE: Half Fourier Single-shot Turbo spin–Echo
TRUFI: True Fast Imaging with Steady-State Free Precession
